# HEMOLYTIC UREMIC SYNDROME ASSOCIATED WITH *STREPTOCOCCUS PNEUMONIAE* IN PEDIATRICS: A CASE SERIES

**DOI:** 10.1590/1984-0462/2020/38/2018065

**Published:** 2019-11-25

**Authors:** Oscar Javier León Guerra, Ricardo Saul Galeano Rodríguez, William Javier Morales Camacho, Jessica Estefanía Plata Ortiz, María Alejandra Morales Camacho

**Affiliations:** a Fundación Hospital de la Misericordia, Bogotá, Colombia.; b Universidad El Bosque, Bogotá, Colombia.; c Universidad Antonio Nariño, Bogotá, Colombia.

**Keywords:** Hemolytic-uremic syndrome, Streptococcus pneumoniae, Renal insufficiency, Síndrome hemolítico-urêmica, Streptococcus pneumoniae, Insuficiência renal

## Abstract

**Objective:**

To describe a case series of four (4) patients with hemolytic uremic syndrome due to *Streptococcus pneumoniae* in a level four complexity institution in the city of Bogotá, D.C., Colombia.

**Cases description:**

We describe cases of four patients who presented respiratory symptoms and fever. All four patients were in regular conditions on hospital admission, after which they required intensive care and ventilatory support. Upon admission, three cases showed evidence of pleuropulmonary complication. Penicillin-sensitive *Streptococcus pneumoniae* was isolated in all cases. All patients presented anemia, severe thrombocytopenia, schistocytes on peripheral blood smear, and hyperazotemia. They required blood transfusion and renal replacement therapy during their hospitalization. The patients were diagnosed with hemolytic uremic syndrome due to *S. pneumoniae*. Three of the four patients had a progressive recovery of the renal function and were discharged after an average of 36 days of hospital stay. The remaining patient had two amputations in the extremities due to thrombotic vascular complications and was discharged after 99 days of hospital stay, requiring hemodialysis every other day.

**Comments:**

Hemolytic uremic syndrome due to *Streptococcus pneumoniae* is a rare but severe complication of invasive pneumococcal disease. Complicated pneumonia is the main condition associated with this entity. It is noteworthy the short period in which these cases were presented, considering the low annual incidence of the disease.

## RESUMO

**Objetivo:**

Descrever uma série de casos de quatro pacientes com síndrome hemolítico-urêmica por pneumococo em uma instituição de referência em Bogotá, Colômbia.

**Descrição dos casos:**

Descrevemos os casos de quatro pacientes que apresentaram sintomas respiratórios e febre. Todos estavam em estado geral regular à admissão hospitalar e necessitaram de cuidados intensivos e suporte ventilatório. Na admissão, em três dos casos foi evidenciada a complicação pleuropulmonar. Isolamento de *Streptococcus pneumoniae* sensível à penicilina foi realizado em todos os casos. Os quatro pacientes precisaram de transfusão sanguínea e terapia de reposição renal durante a hospitalização. Nos testes laboratoriais, observou-se anemia, trombocitopenia grave, presença de esquizócitos em esfregaço de sangue periférico e hiperazotemia. Com esse quadro, o diagnóstico foi de síndrome hemolítico-urêmica associada à infecção por *S. pneumoniae*. Houve recuperação progressiva da função renal em três dos quatro pacientes, que tiveram alta após 36 dias de internação hospitalar, em média. Um paciente teve complicações vasculares trombóticas, resultando em duas amputações nas extremidades, e teve alta após 99 dias de internação, com necessidade de hemodiálise em dias alternados.

**Comentários:**

A síndrome hemolítico-urêmica por *Streptococcus pneumoniae* é uma complicação rara, mas grave, da doença invasiva pneumocócica. A pneumonia complicada é a principal condição associada a essa entidade. Destaca-se o curto período em que esses casos foram apresentados, levando em conta a baixa incidência anual de síndrome hemolítico-urêmica.

## INTRODUCTION

Hemolytic uremic syndrome (HUS) is characterized by the classic triad of microangiopathic hemolytic anemia, thrombocytopenia, and acute renal failure.^1^ More than 90% of the cases occur after an infectious process caused by strains of *Escherichia coli*, which produce the Shiga-like toxin.^1^ *Streptococcus pneumoniae*-associated hemolytic uremic syndrome (Sp-HUS) is an infrequent but severe complication of invasive pneumococcal infection, first described by Fischer et al. in 1971.^2^ It is currently responsible for 5 to 15% of all HUS cases worldwide.^3^ ^,^ ^4^ ^,^ ^5^ The estimated annual incidence is 0.015 cases per 100,000 children between 0 and 17 years of age.^6^ ^,^ ^7^ The clinical course is typically more severe and associated with increased mortality.^1^ ^,^ ^8^ Sp-HUS treatment is primarily supportive through the early administration of antibiotics, blood transfusion, and renal replacement therapy as needed.^1^ ^,^ ^3^ ^,^ ^8^

The purpose of this article is to describe a case series of four patients with HUS due to pneumococcus in a level four complexity institution in the city of Bogotá, D.C., Colombia.

## CASES DESCRIPTION

### CASE 1

A previously healthy 2-year-old male patient, with a 2-day history of fever associated with rhinorrhea and cough and no recent history of travels or contact with infectious diseases. At the physical examination on admission, his condition was regular: tachycardic, tachypneic, with subcostal retractions, and hypoventilation at the right lung base. Laboratory tests showed an absence of leukocytosis or neutrophilia, presence of severe thrombocytopenia (20,000 / mm^3^), markedly elevated C-reactive protein (CRP) (318 mg/L), and negative respiratory viral panel (respiratory syncytial virus - RSV, adenovirus, influenza A and B). Chest x-ray revealed right pneumonia with pleural effusion, leading to the start of antibiotic treatment with ceftriaxone and clindamycin. Subsequent progressive deterioration of his clinical condition required ventilatory and vasopressor support. During his stay in the Intensive Care Unit (ICU), his condition progressed to persistent oligo-anuria associated with hyperazotemia. Renal replacement therapy was started with continuous venovenous hemofiltration. The treatment of anemia and severe thrombocytopenia required multiple transfusions of blood products. Pleural fluid culture was positive for sensitive pneumococcus. Peripheral blood smear showed anisocytosis and schistocytes. The diagnosis was septic shock and multi-organ dysfunction of pulmonary origin due to pneumonia, complicated by Sp-HUS*,* with presence of azotemia, thrombocytopenia, and microangiopathic hemolytic anemia. Lobectomy and decortication were performed due to pleuropulmonary complication. During his stay in ICU, the patient presented infectious deterioration and received meropenem and linezolid. There was a progressive recovery of diuresis with normalization of renal function. He left the institution in good condition, showing recovery of his clinical status about 50 days after admission.

### CASE 2

An 18-months-old male patient without previous diseases presented a 5-day history of cough associated with fever and progressive clinical deterioration and no recent history of travels or contact with infectious diseases. On physical examination, the patient was very irritable, with tachycardia, tachypnea, and diffuse hypoventilation in the left lung field, predominantly at pulmonary bases, requiring supplemental oxygen. Chest x-ray showed multilobe pneumonia. Thoracic ultrasound was compatible with septated empyema in the left hemithorax. On admission, blood count was normal, and the patient presented elevated acute phase reactants (CRP: 326.6 mg/L). He was admitted due to complicated pneumonia and started antibiotic treatment with ceftriaxone and clindamycin. Subsequently, he underwent thoracoscopic decortication, which revealed purulent pleural fluid. Blood cultures were positive for multisensitive *Streptococcus pneumoniae* on admission. About 30 hours after admission, there was progressive deterioration of hemodynamic status, with oliguria, generalized edema, and a trend towards hypotension, despite repeated administration of crystalloids. He was transferred to the ICU with ventilatory, vasopressor, and inotropic support. At this time, the child presented severe anemia (Hb: 4 g/dL), low platelet count (15,000/mm^3^), and hyperazotemia. Peripheral blood smear showed schistocytes. The patient was diagnosed with HUS due to pneumococcal infection associated with multi-organ dysfunction. Renal replacement therapy was started with continuous venovenous hemofiltration. During the hospital stay, he had a complication due to a central catheter-associated infection, which required antibiotic therapy with cefepime after *Enterobacter cloacae* isolation. The patient improved slowly and was discharged after 36 days of hospital stay.

### CASE 3

A 16-year-old male adolescent with a history of Wiskott-Aldrich syndrome and previous splenectomy presented a 2-day history of fever associated with upper respiratory symptoms. He had a recent hospitalization for left basal pneumonia, which was treated with crystalline penicillin. Physical examination showed regular clinical status, hypotension, poor perfusion, tachycardia, and signs of moderate dehydration. He was admitted with the diagnosis of septic shock. Fluid management and antibiotic treatment (ceftriaxone) were initiated. Initial laboratory exams showed metabolic acidosis with elevated lactate levels, mild thrombocytopenia (115.000/mm^3^), and presence of schistocytes on peripheral blood smear. Due to cardiorespiratory failure, the patient required mechanical ventilation and inotropic and vasopressor support. Blood cultures showed a sensitive *Streptococcus pneumoniae*. During his hospital stay, he had multiple complications due to bleeding (upper digestive tract hemorrhage, and pulmonary hemorrhage), requiring repeated transfusions, in addition to progressive hyperazotemia and persistent anuria, needing renal replacement therapy with hemofiltration. Multiple ischemic lesions were documented in the extremities, which progressed to necrosis. He also received plasmapheresis on five occasions. In his clinical context, the diagnosis of thrombotic microangiopathy HUS was considered. ADAMTS-13 levels were measured without adjacent deficit (>5%). After hemodynamic and respiratory stabilization, supracondylar amputation of the left upper limb and right lower transtibial limb was necessary. He was discharged after 99 days of hospital stay, without recovery of renal function, needing hemodialysis every other day.

### CASE 4

A 9-month-old male patient with no significant medical history was admitted with a 5-day history of fever and progressive respiratory distress. He was irritable on admission, with tachypnea, desaturations, and subcostal retractions. Initial laboratory tests showed normal blood cell count; blood gases with mixed respiratory acidosis; elevated acute phase reactants (CRP: 458.6 mg/L); chest x-ray with right multilobar parenchymal involvement associated with pleural effusion, confirmed by ultrasound. Antibiotic coverage was initiated with ceftriaxone and clindamycin. Subsequently, he underwent a right thoracoscopic decortication. Clinical and laboratory controls at 48 hours presented moderate anemia (hemoglobin: 8.3 g/dL), severe thrombocytopenia (17.000/mm^3^), and hyperazotemia, with signs of water overload and persistent oliguria, positive Direct Coombs test, and presence of schistocytes and elevated reticulocytes on blood smear. Due to the development of a digestive tract hemorrhage during his clinical stay, he needed blood transfusion on several occasions. Also, given the worsening renal function tests, renal replacement therapy with peritoneal dialysis was established. Blood cultures on admission revealed a sensitive *Streptococcus pneumoniae*. In the recovery process, thoracotomy revealed purulent secretion, with the reappearance of fever, increased CRP, requiring treatment with carbapenem and linezolid. The patient had a slow clinical improvement with progressive recovery of renal function and hemodynamic stability. He was discharged after 37 days of hospital admission (Table 1).

**Table 1:** Clinical and laboratory characteristics of patients.

Characteristics	Case 1	Case 2	Case 3	Case 4
Age	24 months	18 months	16 years	9 months
Gender	Male	Male	Male	Male
Disease	Pneumonia	Pneumonia	Pneumonia	Pneumonia
Maximum creatinine (mg/dL)	2.69	3.27	8.4	1.57
Maximum AST/ALT (U/L)	502/195	671/189.5	2.363/574.8	67.3/16.6
Minimum hemoglobin (g/dL)	5.6	4.9	5.5	7
Minimum platelets (µL)	18.000	15.000	13.000	12.000
Maximum CRP* (mg/L)	318	326.6	365.5	458.6
Schistocytes on peripheral blood smear	Yes	Yes	Yes	Yes
Direct Coombs test	Positive	Positive	Positive	Positive
Days of dialysis	27	21	>48	10
Type of dialysis	Continuous VV hemodiafiltration	Continuous VV hemodiafiltration	Hemodiafiltration	Peritoneal dialysis
Length of hospital stay	50	36	99	37
Recovery of renal function	Yes	Yes	No	Yes
Extrarenal complications	No	No	Yes**	No
Mortality	No	No	No	No

*Semiquantitative method; **extremity amputation; AST: aspartate aminotransferase; ALT: alanine aminotransferase; CRP: C-reactive protein; VV: venovenous.

## DISCUSSION

Sp-HUS is a rare but severe complication of invasive pneumococcal disease^1^ that mainly affects neonates and children under 2 years of age.^1^ ^,^ ^8^ Despite the severity of the condition, its pathogenesis is still not well defined. It is believed that the neuraminidase produced by *Streptococcus pneumoniae* removes N-acetylneuraminic acid from various glycoproteins and glycolipids on the membrane surface of erythrocytes, platelets, and glomerular capillaries, thus exposing the Thomsen-Friedenreich antigen (T antigen),^3^ ^,^ ^9^ ^,^ ^10^ which reacts to the anti-T antibody, present in most individuals, and initiates the characteristic clinical triad: renal failure, microangiopathic hemolytic anemia, and thrombocytopenia.^3^ ^,^ ^9^ ^,^ ^10^ Additionally, in more than 90% of Sp-HUS cases, the direct Coombs test can be positive due to the binding of anti-T antibodies to recently exposed T antigens on the membrane of red blood cells.^11^ The described mechanisms contribute to the pathogenesis of Sp-HUS. Nevertheless, some authors highlight that host genetic, immune, and environmental factors play an important role in the development of this complication in patients with invasive pneumococcal disease.^9^ Estimates indicate that the annual incidence varies between 0.015‒0.065 cases per 100,000 children aged 0‒18 years.^6^ However, its actual incidence is uncertain since it is believed that this disease has been significantly underdiagnosed, mainly because of the lack of specific tests and well-defined diagnostic criteria, which complicates the adequate differential diagnosis with entities such as disseminated intravascular coagulation^12^ ^,^ ^13^ ^,^ ^14^ that has fibrinogen consumption and abnormalities in clotting times.^15^ One of the striking aspects in our case series is the short interval (<4 months) in which these 4 patients were admitted to the hospital, considering that our institution is a Colombian referral center and a study previously conducted in this hospital with 24 cases of invasive pneumococcal disease over a 5-year period only had one case of Sp-HUS reported.

Some authors include Sp-HUS within what is traditionally known as atypical hemolytic uremic syndrome (aHUS).^16^ ^,^ ^17^ ^,^ ^18^ Nowadays, however, this term tends to be referred to as complement-mediated hemolytic uremic syndrome, characterized by genetic mutations of complement factors. ^16^ ^,^ ^17^ ^,^ ^18^ The most frequent mutations occur at the level of complement factor H (20‒30%), CD46 - formerly known as MCP - (5‒15%), complement factor I (4‒10%), C3 (2‒10%), and complement factor B (1‒4%), among others.^16^ ^,^ ^17^ ^,^ ^18^ Despite the advances in the diagnosis of these mutations, about 40% of aHUS cases have no identifiable abnormality in the complement level.^16^ ^,^ ^17^ ^,^ ^18^

In general, patients with aHUS have a poor prognosis, and it is estimated that more than 60% of patients with some of the previously described mutations die or develop a terminal kidney disease within one year after the onset of the disease.^16^ ^,^ ^17^ ^,^ ^18^ All our patients were treated with the diagnosis of HUS due to pneumococcus. During their hospital stay, no additional studies were carried out to determine genetic mutations in complement factors, so the use of Eculizumab, an anti C5 monoclonal antibody, was not considered. This antibody has clear beneficial effects in the treatment of aHUS, being, therefore, considered the first treatment line.^16^ ^,^ ^17^ ^,^ ^18^

Complicated pneumonia is the main condition associated with Sp-HUS, and might be present in up to 90% of cases.^4^ ^,^ ^19^ ^,^ ^20^ Meningitis is the second most frequent condition and has been reported in up to 29% of cases; however, it has a higher mortality rate than other conditions associated with the development of this disease (37 vs. 2%).^8^ ^,^ ^21^ Despite only around 30% of patients with pneumonia having positive blood cultures,^13^ in all our cases, microbiological isolation of multisensitive *Streptococcus pneumoniae* was successful. In our case series, we highlight the young age, under 2 years old, in three of the patients. Also, all of them had a pneumonic process, and three patients had complicated pneumonia documented on admission, as described in the literature. Historically, Sp-HUS is an entity with higher mortality and morbidity rates compared to the typical HUS triggered by strains of *Escherichia coli* producing Shiga-like toxin, which has a mortality rate and frequency of renal sequelae close to 5%.^1^ ^,^ ^8^ ^,^ ^10^ Mortality rates reported for Sp-HUS reach 12.3%. The literature describes that 10% of patients can progress to end-stage renal disease, and up to 16% of them can develop chronic kidney disease or hypertension.^1^ ^,^ ^4^ ^,^ ^11^ ^,^ ^13^ ^,^ ^21^ Additionally, patients who develop this severe complication tend to be younger and have a greater renal, hematological, and neurological involvement reflected in a longer duration of oliguria, greater need for dialysis therapy and blood transfusion, and longer hospital stays, which increase the risk of unfavorable outcomes and costs to the health system.^1^ ^,^ ^3^ ^,^ ^4^ ^,^ ^15^ All our patients needed an early start of renal replacement therapy and blood transfusion, given their clinical and hemodynamic involvement, as well as their prolonged hospital stays (≥36 days). However, contrary to what was previously reported regarding mortality in the context of Sp-HUS in pediatrics, none of our patients died.

HUS may lead to a series of renal and extrarenal sequelae that can affect the quality of life of pediatric patients. The percentage of patients with long-term sequelae can reach 40%.^8^ One of the most feared comorbidities is the chronic kidney disease, whose main risk factor is the requirement for dialysis therapy for more than 20 days.^4^ In our case series, although three patients had this risk factor, only one of them developed chronic kidney disease and continued with renal replacement therapy. The most common extrarenal complications are pancreatitis, cholecystitis, hepatitis, hearing loss, and extremity amputation;^4^ the latter was observed in Case 3. Although the differential diagnosis with other entities, such as catastrophic antiphospholipid syndrome (CAPS), was difficult in this case due to the lack of additional studies, such as lupus anticoagulant and anticardiolipin antibodies, during hospital stay, our patient had no thrombosis documented in the main organs involved in CAPS (kidney, heart, brain, liver).^22^ ^,^ ^23^ ^,^ ^24^ The multi-system thrombosis in CAPS leads to high morbidity and mortality.^22^ ^,^ ^23^ ^,^ ^24^ Additionally, the severe thrombocytopenia and schistocytosis observed in Case 3 are very rare or absent in CAPS but frequent in HUS.^22^ ^,^ ^25^ We also noted the use of plasmapheresis with 5% albumin in Case 3, based on the possibility of eliminating anti-T IgM antibodies and bacterial neuraminidase,^26^ with good results in the clinical and hemodynamic state of the patient. Nonetheless, some authors do not recommend this therapy, as it can increase pre-existing secondary hemolysis in the presence of anti-T IgM class antibodies in the plasma and result in a reaction with the T antigen exposed in patients with Sp-HUS.^26^ Observations made in vitro indicate that anti-T causes these reactions mainly at temperatures as low as 4°C. The significance of this phenomenon at 37°C (body temperature) has not been documented. The indication of plasmapheresis is currently reported as category III.^13^ ^,^ ^26^ In cases that require additional transfusion of blood products, they should ideally be washed to avoid an increase in levels of preformed anti-T antibodies, which are present in high levels in products that do not receive such intervention.^26^

Since the implementation of the 7-valent pneumococcal conjugate vaccine, cases of invasive pneumococcal disease decreased by about 90% in countries like the United States.^27^ ^,^ ^28^ ^,^ ^29^ However, afterward, the so-called “replacement strains”, such as 3, 6A, 12F, and 19A, which are often resistant to penicillin and other antibiotics, led to the introduction of the 13-valent pneumococcal conjugate vaccine (PCV13) in 2010^27^ ^,^ ^29^ ^,^ ^30^ in order to increase the coverage for different serotypes. Nevertheless, despite these vaccines, new emerging serotypes have been reported, which are not covered by any of the previously mentioned vaccines, such as 15B, 23A, 23B, and 35B.^27^ Ensuring an adequate vaccination program for children is an important tool that does not eliminate the possibility of developing a pneumococcal infection, but dramatically reduces the chance of having an invasive disease or complications associated with this pathology.^27^ ^,^ ^28^ ^,^ ^29^ ^,^ ^30^
